# Psycho-oncology: Mental Illness and Psychopharmacology in Breast Cancer

**DOI:** 10.1192/j.eurpsy.2025.1794

**Published:** 2025-08-26

**Authors:** S. M. Sousa, D. Areias, R. Dionísio, C. Portela, F. Leitão, N. Oliveira

**Affiliations:** 1 Psychiatry, Unidade Local de Saúde de Santo António, Porto, Portugal

## Abstract

**Introduction:**

Patients with breast cancer have a higher incidence of psychiatric symptoms and mental illness compared to those without a cancer history. Breast cancer survivors often experience increased depression, anxiety, neurocognitive and sexual dysfunction, sleep disorders, stress-related conditions, and other psychiatric issues. Psychopharmacological intervention plays a key role in managing both comorbid mental illness and cancer-related symptoms caused by the disease or its treatment. However, there is limited scientific evidence regarding the specificities of psychotropic drug prescriptions in patients undergoing antineoplastic therapy.

**Objectives:**

Considering the specificities of this population (greater physical fragility, polypharmacy) and the limited scientific evidence on the subject, the aim of this work is to review the literature on the use of psychotropic drugs—antidepressants, anxiolytics, antipsychotics, mood stabilizers, and psychostimulants—in the treatment of psychiatric symptoms and cancer-related symptoms in this patient population, with a particular emphasis on psychopharmacological-antineoplastic drug interactions.

**Methods:**

A non-systematic literature review using PubMed as the database.

**Results:**

In patients undergoing tamoxifen treatment, the use of psychotropic drugs that have inhibitory properties on cytochrome P450 (CYP2D6), the enzyme responsible for its metabolism into its active form (endoxifen) should be avoided. This specificity limits the use of strong inhibitors of this enzyme, such as some selective serotonin reuptake inhibitors—fluoxetine, paroxetine, and fluvoxamine. In the use of antipsychotics, the preference for drugs less associated with hyperprolactinemia should be considered when managing these patients. Commonly used mood stabilizers, such as lithium and valproic acid, do not interact with the most commonly used antineoplastic drugs in breast cancer, and are frequently used in this population safely, requiring the same monitoring as in the general population.

**Conclusions:**

The need for pharmacological management of psychiatric symptoms or illnesses in the breast cancer population is a reality for which there is still little information in the available scientific literature. It is crucial that the use of psychotropic drugs is carefully monitored due to the potential pharmacological interactions with antineoplastic treatments, which may alter the efficacy or increase the toxicity of the drugs. For this reason, the present review is important and can enrich clinical practice. Collaboration between psychiatrists and oncologists is essential to optimize treatment and improve the quality of life for these patients.

**Disclosure of Interest:**

None Declared

